# Impact of a fixed price system on the supply of institutional long-term care: a comparative study of Japanese and German metropolitan areas

**DOI:** 10.1186/1472-6963-14-48

**Published:** 2014-02-01

**Authors:** Keiko Yoshida, Kazuo Kawahara

**Affiliations:** 1 Department of Health Policy Science, Tokyo Medical and Dental University, Graduate School of Medical and Dental Science, 1-5-45 Yushima, Bunkyo-ku, Tokyo 113-8519, Japan

**Keywords:** Long-term care, Institutional care facility, Excess demand, Equitable supply, Fixed price system, Metropolitan areas

## Abstract

**Background:**

The need for institutional long-term care is increasing as the population ages and the pool of informal care givers declines. Care services are often limited when funding is controlled publicly. Fees for Japanese institutional care are publicly fixed and supply is short, particularly in expensive metropolitan areas. Those insured by universal long-term care insurance (LTCI) are faced with geographically inequitable access. The aim of this study was to examine the impact of a fixed price system on the supply of institutional care in terms of equity.

**Methods:**

The data were derived from official statistics sources in both Japan and Germany, and a self-administered questionnaire was used in Japan in 2011. Cross-sectional multiple regression analyses were used to examine factors affecting bed supply of institutional/residential care in fixed price and free prices systems in Tokyo (Japan), and an individually-bargained price system in North Rhine-Westphalia (Germany). Variables relating to costs and needs were used to test hypotheses of cost-dependency and need-orientation of bed supply in each price system. Analyses were conducted using data both before and after the introduction of LTCI, and the results of each system were qualitatively compared.

**Results:**

Total supply of institutional care in Tokyo under fixed pricing was found to be cost-dependent regarding capital costs and scale economies, and negatively related to need. These relationships have however weakened in recent years, possibly caused by political interventions under LTCI. Supply of residential care in Tokyo under free pricing was need-oriented and cost-dependent only regarding scale economies. Supply in North Rhine-Westphalia under individually bargained pricing was cost-independent and not negatively related to need.

**Conclusions:**

Findings suggest that publicly funded fixed prices have a negative impact on geographically equitable supply of institutional care. The contrasting results of the non-fixed-price systems for Japanese residential care and German institutional care provide further theoretical supports for this and indicate possible solutions against inequitable supply.

## Background

In OECD countries, the proportion of adults aged 65 years and over is projected to grow in the next 4 decades [1]. The increase in the very old population causes particular concern about availability of informal care, and future supply shortages of institutional care. In Japan, the proportion of older persons reached 23% in 2010 and is projected to increase further [2]. To avoid higher costs, the Japanese government is trying to promote services in the home care setting including residential care (e.g. private nursing homes, PNHs), rather than expanding institutional facilities (IFs) [3]. It is however uncertain whether the promotion of home care reduces the likelihood and necessity of entering into IFs [4], especially because IF residents are very old, highly dependent, and need intensive care. Furthermore, it is expected that fewer family caregivers will be available in the future [5,6].

The supply of beds in IF is neither sufficient nor evenly distributed in Japan [7,8], although the long-term care insurance (LTCI) law implies that municipalities and prefectures should arrange for a need-oriented provision of care services within the municipality and neighboring municipalities [9]. They should enable the elderly to remain in their own communities with their family, social networks and familiar surroundings. This is consistent with the ‘aging in place’ concept, which has recently gained international recognition [10,11]. However, admission to an IF usually requires a long period of waiting. In 2011, the ratio of eligible adults waiting for a bed to those available was 3:1 in special nursing homes for the elderly (SNHE), a primary type of IF. The ratio was even higher in metropolitan compared with rural regions [12].

According to health policy research, shortage of medical or care services often occurs when funding is controlled publicly, as cost efficiency and containment are likely priorities [13]. Furthermore, economic theory states that the quantity demanded exceeds the quantity supplied if the price is fixed below the equilibrium e.g. [14,15]. The higher costs in metropolitan areas dissuades suppliers to establish IFs as the gap between overall uniformly fixed price and equilibrium prices is larger than in rural areas (Additional file 1).

Excess demand resulting from low fixed fees tends to remain stable, as described by Scanlon [16]. Scanlon’s model^a^ explaining excess demand in the US state-regulated nursing home market (under fixed prices) can also be partly applied to Japan: the Japanese government, as the dominant purchaser, tends to set remuneration below the market price. Higher fees would be needed to incentivize more suppliers to establish IFs. However, the government typically does not increase fees to avoid an increase in use. Institutional care is more heavily subsidized and therefore less expensive for users than other corresponding services. As such, raising fees does not reduce demand. More users of IFs imply a larger share of the most costly care—LTC services. As a result, excess demand is economically optimal for the paying authorities, but conflicts with their political goal.

In political discussions the shortage is often attributed to disproportionately high cost or low remuneration. Some Japanese studies and reports found that shortage of appropriate land and high prices negatively affect bed capacity in institutional care e.g. [8]. In the USA, where there was excess demand for subsidized institutional care, previous studies showed a negative effect of low reimbursement rates on bed supply [17,18]. Theoretically, high costs or low remunerations constrain profit, and thus supply, particularly under fixed pricing. However the price system itself has seldom been analyzed as an institutional cause of shortage in the field of LTC.

In the hospital sector, fixed pricing (a type of prospective payment system) had replaced cost-based pricing in the USA and several other countries to enhance cost-efficiency. The effects of this change have been intensively investigated. Besides the expected effects on costs, a negative impact on supply/access has been reported: smaller rural hospitals in the USA were closed, because the fixed remuneration for publicly subsidized cases (Medicare patients) was too low [19-21]. They were not able to achieve a profit because of low resident volume and high average costs.

The aim of this study is to empirically examine the relationship between the price system and supply of institutional elderly care. Based on economic theory and modeling, this study specifically asks whether cost–dependency of supply and therefore inequitable supply is caused by publicly fixed pricing.

## Methods

### Design

This study consists of multiple case studies that were each analyzed cross-sectionally [22]. The relationship between supply and factors affecting supply of IFs under fixed pricing in Tokyo was compared with two non-fixed price systems: a system of free prices and of prices bargained based on facilities’ individual costs. The PNH market in Tokyo (Japan) was analyzed as sample for the former and the IF market in North Rhine-Westphalia (NRW, Germany) for the latter [23,24]. These two markets have grown under LTCI so far that no excess demand is seen at least in aggregated quantity. The average bed occupancy rate of Japanese PNHs amounted to 89% in 2007^b^, and IFs in NRW declined from 98% in 1998 to 89% in 2009 [25-27].

The comparison procedure was conducted in three steps. First, institutional and geographical characteristics were described focusing on factors related to supply. Second, the relationship between supply and factors possibly affecting supply was statistically analyzed for each market separately, as no direct quantitative comparison of the data was possible. Third, factors affecting supply were compared qualitatively among the three systems.

### Institutional background for comparison

Japan (2000) and Germany (1994) introduced LTCI based on a mandatory social insurance model. Historically, institutional elderly care was predominantly tax-funded and publicly planned in both countries, and provided mainly by private not-for-profit organizations [24,28-30]. Eligibility was decided by the local authorities (and facilities in Germany [23]). After introducing LTCI, both countries aim to promote a market-oriented provision of services to allow the insured to choose services and provider [3,24,31]. The regulatory frameworks for three facility types considered as relevant to supply behavior are described in Table 1. In summary, Japanese IF market is more strongly regulated and less market-based, and its prices are less cost-based than those of Japanese PNHs and German IFs.

**Table 1:** Relevant regulatory differences among three facility types

	**Institutional facilities (Japan)**	**Private nursing homes (Japan)**	**Institutional facilities (Germany)**
Market entrance	-Supply control: authorities can reject approvals in terms of their referring levels capping bed density [28] -Service provision planning by each municipality as insurer & prefecture	-Not subject to authorities’ provision planning (-2006)	-No supply control under LTCI: LTCI funds must contract with any provider who meets quality standard [24].
		-No supply control (-2006)	-Need planning: abolished
Providers	-Not-for-profit; small minority is municipal	- Majority is for-profit	-Mixed: for-profit & not-for-profit; minority is municipal
Financing LTCI	-Half by contributions & half by taxation [32] (Public payers: state, prefecture & municipality)	-Half by contributions and half by taxation [32] (Public payers: state, prefecture & municipality)	-Financed solely by contributions
Financing care fees	-Fees: publicly fixed	- Fees: publicly fixed under LTCI	-Before LTCI: fully financed based on cost- recovery-principle [23]
	-Co-payment before LTCI: according to ability-to- pay principle	-Different according to eligible levels	-Fees: bargained between providers & social-assistance (SA) sponsor/LTCI funds etc.; reflecting facility’s individual costs for each eligible level
	-Co-payment under LTCI: 10% of fees	- Co-payment: 10%	-Benefits under LTCI: publicly fixed and capped, according to eligible levels
	-Different according to eligible levels under LTCI	-Care fees for PNHs are lower than that for IFs	-Users who cannot co- payment: eligible for SA
Financing hotel costs (accommodation & meals)	-Fees: Publicly fixed; not adjusted to local price/rent level	-Fees: market-based; set by facility	-Before LTCI: fully financed based on cost- recovery-principle [23]
	- Co-payment: depending on room type and income	-Fully paid out-of-pocket	-Fees: individually bargained between LTC fund and facility (provider) based on costs [33]
	-After 2005, middle and high income users are no longer subsidized & pay full price set by facility out-of-pocket	-Not subsidized	-Fully paid out-of-pocket -Users who cannot co- payment: eligible for SA
	-Users who cannot co-payment eligible for SA		
Interests of insured persons	Preferable to other residential facilities due to higher subsidies, not-for-profit status & no time-limits [34]	More expensive alternative to IFs	-SA-recipients: preferable to other services due to higher subsidies
			-Not-SA-recipients: more expensive than home care due to considerable out- of-pocket payments [24]
Interests of authorities (J) & LTCI funds (G)	Economic incentive to constrain IF supply & fees, but politically for need-oriented provision	Economically preferable due to lower benefits & almost no subsidy compared to IFs	No strong economic incentive to constrain fees & expenditures
Subsidy for (initial) capital costs	- Amount is based on a national standard [28]	Rarely	-Before LTCI: directly paid (only to not-for-profit facilities)
	-Paid directly by state		-Under LTCI: capital costs are fully financed by users separately from hotel costs; low- income users are subsidized for capital costs
	-Sometimes additionally subsidized depending on municipal decision		
	-Land acquisition subsidized decreasingly		
Significance	-24% of LTC beneficiaries use IFs (2009; calculated based on official data [35])	-4% of LTC beneficiaries (2009; incl. PNH-similar facilities; calculated based on official data [35])	-30% of LTC beneficiaries (2009) [27]
		-LTCI gave a boost in development of PNHs	
Clientele with LTCI benefits	- Eligible persons assessed as heavily independent	All LTC eligible persons	-All LTC eligible persons
	- Low-income users as majority		- SA-recipients: about 80/30% of users (before /under LTCI) [24,36]

### Geographical background for comparison

Tokyo and NRW are ideal samples to examine effects specific to metropolitan areas. They are both the most highly populated metropolitan areas in their country, with approximately 10% to 20% of the nationwide population, containing a metropolitan core and adjoining rural areas. The Rhine-Ruhr region in NRW is regarded as the sole megacity in Germany with approximately 10 million inhabitants, corresponding to approximately 9 million inhabitants in 23 special wards in the center of Tokyo.

### Conceptual model

The supply of IF depends on investment decisions of potential suppliers, as LTC is primarily privately provided. Accordingly, supply may be affected by the following factors, on the basis of economic theory [14,37,38]: production cost, alternative production, techniques, input substitution, the market for input, remuneration methods, incentives, the number of competing sellers, and demand (including health-related care need).

The main factors of interest in this study were cost and need which varied by region. Production and opportunity costs and market for input were regarded as cost factors. Input substitution was irrelevant to this study, therefore not considered. According to economic theory, high costs constrain the profit, and thus supply, particularly under fixed pricing. Higher need/demand leads to more supply under flexible pricing [14], but not under fixed pricing.

In these analyses, the number of competitors and incentives were considered as factors which could both negatively and positively influence profit [14], particularly under fixed pricing. Both can also be considered political interventions under authorities’ control. Factors which were thought to relate to profit, apart from cost and demand-related factors, were included in analyses as control variables. Techniques, and remuneration methods are likely to be constant within Tokyo and NRW, and therefore were not included in the analyses.

### Hypotheses

Cost-dependency and the equitable distribution of supply were examined in each system to test two hypotheses:

(Hypothesis 1) In fixed price systems, bed supply is negatively related to costs and not positively related to LTC need.

(Hypothesis 2) In free price systems, and system using individually bargained prices, bed supply is not related to costs but is positively related to LTC need.

Cost-dependency refers to a negative relation between costs and bed supply. Equity was defined as need-oriented based on the concept described by Andersen and Newman [39]: when supply is equitable, variables related to care need are expected to have more of a positive impact on supply than socioeconomic variables.

### Data sources

Data on the facilities in Tokyo compiled from official data were provided by the Welfare and Medical Service Agency. Data on demographic and socioeconomic characteristics, and care need were derived from official statistics departments of the Tokyo and Japanese government [40,41]. Data on wages and municipal subsidies were collected by the authors as described below. Data on the facilities, sociodemographic characteristics and care need in NRW were derived from official statistics of the German Federal Statistical Office, and the Information und Technik NRW (State Statistical Office) [42,43]. Data on labor costs and land price are described below.

### Dependent variables

In this study, the supply of/access to LTC facilities was measured by bed density—the total number of beds per 1000 elderly persons (65 years or older) in each municipality [41-43]. Municipalities are insurers of LTCI and responsible for planning the provision of care services in Japan, and thus used as unit of the analyses. In total, 56 wards, cities, towns, villages or sub-prefectures (for small islands) were included into the analysis for Tokyo, and 54 counties and independent cities for NRW.

The occupancy rate, which is often used by authorities as indicator for bed availability in a facility or area, was not used for this study, as it does not provide enough information about equitable access, i.e. the extent and distribution of bed (under-)supply/availability per elderly inhabitant. The occupancy rate refers to actual utilization which depends on e.g. demand [44].

Japanese IFs contain SNHE and health services for the elderly (HSFE). HSFE offers more medically oriented services than SNHE. Eligible service users waiting for admission to SNHE typically stay transitionally at HSFE. Sanatorium-type facilities are the third type of IF but were not considered in these analyses, as they will soon be abolished following a government decision. PNHs approved as LTCI providers of residential care services were included in the analyses, and considered as a for-profit equivalent to IFs. The German IFs contains all nursing homes approved by LTCI funds.

### Explanatory variables

Costs (IFs and PNHs in Tokyo): Cost for land acquisition is the most regionally varying item in the initial capital costs. Thus, land price was used as an indicator of production and opportunity costs, and market for input factor. For Tokyo, land price was measured by the average price of 1 m^2^ in residential areas in each municipality [45].

Wages, the greatest component of operating costs, were used as an indicator of further production cost. As no official wage statistics were available at the municipal level, job advertisements within the public employment office [46] were collected from June to October 2011, and data on full time nurses’ salaries were determined. The average salary was then calculated for each municipality.

Facility size is expected to influence average and marginal costs, as previous studies have suggested that economies of scale exist in the nursing home sector e.g. [47,48]. Facility size was measured by average bed capacity of facilities in each municipality. It was thought that spatial availability motivates suppliers to build a large facility instead of multiple smaller facilities if suppliers expect economies of scale.

Costs (NRW): As data on land price were not available at the municipal level, rent levels were used. Rent levels are published by the German Federal Ministry of Construction and used to indicate average land price in each municipality. Rent levels were provided in six levels, which correspond to the percentage deviation of local rent from the national average rent for comparable housing space [49]. The range of each level (10%) is constant and thus can be treated as interval scale required for regression analyses. Levels 1 and 6 could however be wider than 10% and are potentially underestimated.

Data on wages at the municipal level were also unavailable. Therefore, average time taken to find a skilled elderly care nurse was used to indicate market for input (labor). These data were compiled by the labor employment office for NRW [50], and captures regional under- and over-supply of elderly care nurses which in turn indirectly affects wage level.

Facility size was measured by the average bed capacity of facilities in each municipality [42,43].

Need: Functional impairments in activities of daily living are a strong predictor of utilizing institutional care need [51,52] and determine eligibility for LTCI. The proportion of elderly persons who were assessed as eligible for LTCI was thus used to measure current need [41-43]. In addition, average yearly growth rate was used to assess the dynamics of growth in need. Eligibility data were available only after introducing LTCI [41-43].

Control variables – profit and political intervention: For Tokyo, the number of IF beds per 1000 elderly persons which were subsidized for land acquisition or funded by the municipality was used as an indicator of profit and political intervention. Postal self-completion questionnaires were sent to all municipalities in Tokyo in July 2011 to determine facility names, the number of beds, and the number of years they were supported outside of the prefectural framework. The validity of the questionnaire was piloted in two municipalities. In case of no response after two months, the person in charge of the municipality was contacted. A total of 47 municipalities out of 56 responded (84%). In addition, the number of beds belonging to facilities for which municipalities offered ex-public space were counted using documents issued by the government of Tokyo. Subsidies were not measured for PNHs in Tokyo and IF in NRW, because they are rarely (PNHs) or no longer (NRW) granted. Furthermore, official data on subsidies in NRW from early years were unavailable.

For the numbers of competitors, the initial and average bed densities in the previous years of IFs and PNH were used for the analysis period under LTCI [40-43]. These variables also function as political intervention under supply control for IFs in Tokyo.

Control variables—demand: Apart from care need, the following variables which could induce demand were included in multiple regression models, based on the behavioral model of health care use [39]:

- the proportion and average yearly growth rate of elderly persons to reflect the predisposition of the individual to use services [40-43]

- average residential tax, as a substitute for income (Tokyo) [40] or average disposable income (NRW) [43] to reflect financial ability to secure services.

### Analytical models and regression analysis

Ordinary least squares linear regression analyses were performed for each analysis period with supply of IF beds as the dependent variable for both Tokyo and NRW. Variables were added to these models in a series of steps. First, a univariate regression was conducted for the total bed supply with a health-related care need variable to describe the grade of need-orientation of bed distribution (equation 1). Second, cost- and need-relevant variables were included in addition to a set of control variables in order to examine hypotheses in terms of supply behavior (equation 2). Third, change in bed density was used as the dependent variable. In this model, bed density of IFs shortly before introducing LTCI was also included to adjust for the effect of previous supply (equation 3):

(1) BDi=βYi+α+e

(2) BDi=βXi+βYi+βZi+α+e

(3) △BDi=βBDi0+βXi+βYi+βZi+α+e

(BD_i_ = recent bed density; X_i =_ costs; Yi = need; Zi = control variables which vary by municipality; β = coefficients; α = a constant; e = an error term with a mean of 0; △BDi = change in bed density).

As there were only a small number of cases, variables with low predictive power were excluded from regression models. The combinatorial method was used to identify the combination of variables with the largest adjusted R^2^ for each model size. Among these combinations, the model with the highest adjusted R^2^ value was selected. The adjusted R^2^ is a measure incorporating a trade-off between goodness-of-fit and the number of explanatory variables [53]. Alternatively, model selection using backwards selection in combination with Akaike Information Criterion was also conducted, and identical results were found.

White’s test and variance inflation factors were calculated to check for heteroskedasticity and multicollinearity. Where the null hypothesis of no heteroskedasticity was rejected at the 5% significance level, robust standard errors were applied. The second best model was selected if the first model contained variables with a high degree of multicollinearity (variance inflation factor >10 in one case). EViews 7 was used for all statistical analyses.

### Analysis period

In the first and second equation, distribution and overall supply refer to the bed density of each municipality in 2010 for IFs in Tokyo, and 2009 for IFs in NRW. For PNHs in Tokyo, distribution and overall supply in 2008 were analyzed, reflecting a 2-year lag after introduction of supply control of PNHs in 2006. In the third equation, supply under LTCI refers to change in bed density from 1999 to 2009 for IFs in NRW, and from 2002 to 2010 for IFs in Tokyo. Change in bed density in Tokyo was analyzed only after 2002 to account for a possible time lag in the institutional changes brought by LTCI. As data of NRW are available only after 1999, the lag is automatically accounted for. Japanese PNHs were not analyzed as most PNHs were built after 2000, and overall supply was comparable to supply under LTCI.

Most explanatory variables used were assessed at the time of investment decisions, and not at the time of facility establishment. It takes 2–3 years to set up a facility. Hence, a 2-year lag between decision and supply was considered and the average available years were used to capture long-term trends. In Japan, the development of IFs started after 1963 and accelerated in the 1990s [29]; in Germany, around half were built before 1990. If old data were not available, data from an adjacent year were used to determine quasi-median data. As such, the relationships observed were assumed to be constant between municipalities over the analysis period.

## Results

Tables 2 and 3 show descriptive statistics. Tables 4 and 5 show the results of regression analyses of distribution and supply. In Table 6, hypothesized and analyzed results are compared.

**Table 2:** Summary statistics for equation 1 and 2* comparing 56 municipalities in Tokyo and 54 municipalities in North Rhine-Westphalia (NRW)

***Variables***	***IF in Tokyo***		***PNH in Tokyo***		***NRW***	
	**Mean**	**(Standard deviation)**	**Mean**	**(Standard deviation)**	**Mean**	**(Standard deviation)**
***Dependent variables***						
Overall bed-density^a^	34.82	(41.53)	7.48	(6.15)	47.46	(6.72)
***Explanatory variables***						
**Costs/cost relevant**						
Average facility size (beds)	92.08	(18.28)	61.41	(20.27)	77.3	(14.74)
LTA land price for T (¥1000 s)/LTA rent-level for N	382.54	( 249.39)	336.49	(254.12)	3.11	(1.02)
LTA wage of elderly care nurse for T (¥1000 s)/LTA duration for care nurse search for N^b^ (days)	205.05	(8.92)	205.05	(8.92)	54.3	(15.08)
**Need**						
LTA percent elderly^c^ needing LTC	13.04	(1.97)	15.48	(1.96)	15.7	(2.07)
LTA growth rate of elderly^c^ needing LTC	-	-	8.65	(2.52)	-	-
**Profit/political intervention (control variables)**						
LTA bed-density^a^ of IF	-	-	24.14	(19.98)	-	-
Density^a^ of subsidized beds	5.71	(6.77)	-	-	-	-
**Demand (control variables)**						
LTA percent elderly^c^	14.09	(3.86)	18.12	(4.46)	15.9	(1.59)
LTA growth rate of elderly^c^	4.61	(1.83)	4.15	(1.95)	1.31	(0.67)
LTA residential tax for T (¥1000 s)/LTA disposable income for N (€1000 s)	107.38	(53.60)	107.16	(60.78)	14	(1.51)
N	54-56		47-56		54	

^a^Bed density: number of beds per 1000 elderly adults aged 65 years and over.

^b^Vacancy duration for search of skilled care: time taken for an employer to find a skilled elderly care nurse.

^c^Elderly persons: those aged 65 years and over.

*Equation 1. BDi = βYi + α + e; Equation 2. BDi = βXi + βYi + βZi + α + e; BDi: overall supply in municipality i ; β: coefficients; α: constant; e: error with zero mean; X: variables for costs; Y: variables for need; Zi: control variables.

**Table 3:** Summary statistics for equation 3* comparing 56 municipalities in Tokyo and 54 municipalities in North Rhine-Westphalia (NRW)

***Variables***	***IF in Tokyo***		***IF in NRW***	
	**Mean**	**(Standard deviation)**	**Mean**	**(Standard deviation)**
***Dependent variables***				
Change in bed-density^a^	-0.95	(9.56)	-0.64	(5.89)
***Explanatory variables***				
**Costs/cost relevant**				
Average size of new facilities (beds)	95.54	(26.98)	89.7	(67.55)
LTA land price for T (¥1000 s)/LTA rent-level for N	341.03	(285.98)	3.11	(1.02)
LTA wage of elderly care nurse for T (¥1000 s)/LTA duration for care nurse search for N^b^ (days)	205.05	(8.92)	54.3	(15.08)
**Need**				
LTA percent elderly^c^ needing LTC	15.70	(1.94)	14.5	(1.87)
LTA growth rate of elderly^c^ needing LTC	7.01	(2.21)	2.89	(5.24)
**Profit/political intervention (control variables)**				
Initial bed-density^a^ of IF	32.21	(43.59)	48.3	(8.79)
LTA bed-density^a^ of PNH	3.95	(4.11)	-	-
Density^a^ of subsidized beds	1.45	(2.94)	-	-
**Demand (control variables)**				
LTA percent elderly^c^	18.12	(4.46)	18	(1.50)
LTA growth rate of elderly^c^	4.16	(1.95)	2.04	(0.76)
LTA residential tax for T (¥1000 s)/LTA disposable income for N (€1000 s)	117.63	(64.52)	17.7	(1.72)
N	46-56		50-54	

^a^Bed density: number of beds per 1000 elderly adults aged 65 years and over.

^b^Vacancy duration for search of skilled care: time taken for an employer to find a skilled elderly care nurse.

^c^Elderly persons: those aged 65 years and over.

*Equation 3. Supply under LTCI: ΔBDi = βBDi0 + βXi + βYi + βZi + α + e. ΔBid: change in bed density between the year shortly after LTCI introduction and the recent year in municipality i; BDi0: bed-density shortly before introducing LTCI in municipality i; β: coefficients; α: constant; e: error with zero mean; X: variables for costs; Y: variables for need; Z: control variables.

**Table 4:** Relationship between LTC need and bed density using linear regression

***Explanatory variables***	***Dependent Variables***		
	**Overall bed-density**^**a **^**of IF 2010 Japan**	**Overall bed-density**^**a **^**of PNH 2008 Japan**	**Overall bed-density**^**a **^**of IF 2009 Germany**
	**Coefficient (95**% **CI)**	**Coefficient (95**% **CI)**	**Coefficient (95**% **CI)**
Constant	149.55 (80.55; 218.55)	-1.30 (-14.46;11.85)	25.34 (12.42; 38.26)
**Need**			
LTA percent of elderly^b^ needing LTC	-8.80 (-14.03;-3.56)	0.57 ('-0.28; 1.41)	1.41 (0.59; 2.22)
R2	0.17	0.03	0.19
Adjusted R2	0.15	0.01	0.17
F	11.36^**^	1.81	12.00^**^
N	56	56	54

*p < 0.05; **p < 0.01; ***p < 0.001.

^a^Bed-density: number of beds per 1000 elderly adults aged 65 and over.

^b^Elderly persons: those aged 65 and over.

**Table 5:** Relationship between relevant supply factors and bed density using linear regression

***Explanatory variables***	***Dependent variables***									
	**Overall bed-density**^**a **^**of IF 2010 Tokyo**		**Change in bed-density**^**a **^**of IF 2002-2010 Tokyo**		**Overall bed-density**^**a **^**of PNH 2008 Tokyo**		**Overall bed-density**^**a **^**of IF 2009 NRW**		**Change in bed-density**^**a **^**of 1999 -2009 NRW**	
	**Coefficient (95% CI)**	**β**	**Coefficient (95% CI)**^**b**^	**β**	**Coefficient (95% CI)**	**β**	**Coefficient (95% CI)**	**β**	**Coefficient (95% CI)**	**β**
Constant	-27.85 (-211.13; 155.43)	-	22.69 (0.44; 44.94)	-	43.80 (-3.66; 91.26)	-	32.55 (15.18; 49.91)	-	39.36 (26.61; 52.10)	-
***Explanatory variables***										
**Costs/cost relevant**										
Average facility size (beds)	0.47 (0.03; 0.92)	0.20^* c^	0.03 (-0.02; 0.09)	0.10	0.11 (0.04; 0.18)	0.39^** c^	-0.07 (-0.19; 0.05)	-0.15	Eliminated	-
LTA land price (¥1000 s)/ LTA rent-level for N	-0.08 (-0.15;-0.02)	-0.47^*^	Eliminated	-	-0.01 (-0.02;0.00)	-0.23	Eliminated	-	Eliminated	-
LTA wage of elderly care nurse for T (¥1000 s)/ LTA duration for care nurse search^d^ for N (days)	0.22 (-0.59; 1.04)	0.05	Eliminated	-	-0.19 (-0.37;-0.01)	-0.30^*^	Eliminated	-	Eliminated	-
**Need**										
LTA percent elderly^e^ needing LTC	-8.96 (-13.31;-4.61)	-0.37^***^	-66.26 (-160.91; 23.40)	-0.14	1.16 (0.04; 2.28)	0.32^*^	1.46 (0.63; 2.28)	0.45^***^	Eliminated	-
LTA growth rate of elderly^e^ needing LTC	-	-	-0.82 (-1.87; 0.22)	-0.14	0.88 (0.03;1.74)	0.25^*^	Eliminated	-	Eliminated	-
**Profit/political intervention**										
Initial bed-density^a^ of IF	-	-	-0.21 (-0.30;-0.13)	-0.82^***^	-	-	-	-	-0.39 (-0.52;-0.26)	-0.59^**^
LTA bed-density of IF for PNH/LTA bed-density of PNH for IF in T	-	-	Eliminated	-	Eliminated	-	-	-	-	-
Density of subsidized beds^a^	-0.64 (-1.99; 0.72)	-0.10	0.52 (0.29; 0.76)	0.19^***^	-	-	-	-	-	-
**Demand**										
LTA percent elderly^e^	6.96 (5.15; 8.77)	0.62^***^	Eliminated	-	-1.26 (-2.17;-0.36)	-0.38^**^	Eliminated	-	Eliminated	-
LTA growth rate of elderly^e^	Eliminated	-	-1.09 (-1.94;-0.23)	-0.20^*^	-0.99 (-2.31; 0.34)	-0.29	-2.20 (-4.86; 0.48)	-0.22	- 1.85 (-3.36;-0.35)	- 0.24^*^
LTA residential tax for T (¥1000 s)/LTA disposal income for N (€1000 s)	0.25 (-0.02; 0.52)	0.32	Eliminated	-	Eliminated	-	Eliminated	-	-0.98 (-1.64;-0.31)	-0.29^**^
R2	0.74	-	0.82	-	0.56	-	0.24	-	0.56	-
Adjusted R2	0.70	-	0.79	-	0.48	-	0.19	-	0.53	-
F	18.70^***^	-	29.92^***^	-	7.17^***^	-	5.13^**^	-	20.82^***^	-
N	54	-	51	-	47	-	54	-	54	-

*p < 0.05; **p < 0.01; ***p < 0.001.

^a^Bed density: number of beds per 1000 elderly adults aged 65 years and over.

^b^Robust standard errors were computed to correct for possible heteroskedasticity.

^c^Both coefficients for average facility size remained positively significant after additional adjustment for the number of elderly adults accounting for bias caused by bed density which automatically increases with decreasing number of elderly adults.

^d^Vacancy duration for search of skilled care nurse: time taken for an employer to find a skilled elderly care nurse.

^e^Elderly persons: those aged 65 years and over.

**Table 6:** Hypothesized and observed relationships between supply and cost/need relevant variables

***Explanatory variables***	***Dependent variables***							
	**Bed-density of IF Tokyo**			**Bed-density of PNH Tokyo**		**Bed-density of IF NRW**		
	**Hypothesized**	**Result: overall**	**Result: change**	**Hypothesized**	**Result: overall**	**Hypothesized**	**Result: overall**	**Result: change**
**Costs/cost relevant**								
Average facility size	+	+	0	0	+	0	0	0
LTA land price	-	-	0	0	0	0	0	0
LTA wage	-	0	0	-	-	0	0	0
**Need**								
LTA percent elderly needing LTC	0/-	-	0	+	+	+	+	0
LTA growth rate of elderly needing LTC	0/-	0	0	+	+	+	0	0

NOTE:

+: Positive relationship.

-: Negative relationship.

0: No significant relationship.

### Description of equity/inequity in distribution

The standard deviation of overall densities of IF beds in Tokyo (41.53) was larger than for NRW (6.72), i.e. a more uneven bed distribution in Tokyo (Table 2). The univariate regression results (Table 4) showed negative associations between need (proportion of elderly needing LTC) and bed density of IFs for Tokyo (2010), and a positive association with NRW (2009) at the 5% and 1% significance-level. Need was not bivariately associated with bed-density of PNH in Tokyo (2008).

### Supply analysis

**IFs in Tokyo (Table** 5** and **6**)** Overall supply: The two cost-relevant variables (land price and facility size) were significantly (at the 5% level) related to bed density in the expected directions, i.e. higher land price and poor spatial availability were associated with lower bed density. Wages were not significant associated with bed density.

As hypothesized, need (the proportion of elderly needing LTC) was not positively related to bed density**—**it was negatively related at the 0.1% level. The need variable growth rate of needy elderly was not significantly related to bed density. Among the control variables, only the proportion of elderly persons was positively related to supply (at the 0.1% level). Contrary to general expectation, this variable for aging was not positively associated with the need variable (Additional file 2).

Supply under LTCI: Results only partially supported the first hypothesis. As hypothesized, need-variables and change in supply were not significantly related, with negative coefficients. However, supply under LTCI was unexpectedly not related to costs. Control variables for profit and political intervention (lower initial bed density and higher subsidy) were significant related to higher bed density (at the 0.1% level). The control variable average growth rate of elderly persons was negatively related to change in supply (at the 5% level), i.e. additional bed density was lower in municipalities where elderly population grew faster. All other control variables were not significantly related to change in bed density.

**Overall supply of PNHs in Tokyo (Table **5** and **6**) **Unexpectedly, facility size (a cost-related variable) showed a significantly positive association with PNH supply under free pricing (at the 1% level). The bivariate correlation between facility size and land price was however not significant, unlike IFs, i.e. PNH suppliers tend to look for larger space independently of land price (Additional file 2 and Additional file 3). As hypothesized, land price was not related to supply. Wages were unexpectedly negatively associated with supply at the 5% significance level. This is not consistent with the second hypothesis but with the first hypothesis, as wages are covered by fixed care fees. As hypothesized, the need-related variables growth rate of elderly persons needing LTC and the proportion of elderly persons needing LTC were positively related to bed density (at the 5% significance level). More PNH beds were supplied in municipalities where more eligible elderly persons for LTC live, and PNH beds tended to increase in areas with greater growth rates of eligible persons. The control variable for aging (the proportion of elderly persons) was significantly negatively associated with bed density (at the 1% level), suggesting that bed supply had not caught up with the extent of aging.

**IFs in NRW (Tables **5** and **6**)** Overall supply: The proportion of elderly persons needing LTC was the only variable which was significantly positively related to bed density in 2009 (at the 0.1% level). This indicates a need-oriented supply of IFs in total, and no cost-dependency as hypothesized.

Supply under LTCI: As hypothesized, supply was not related to any of the cost-relevant variables. Unexpectedly, need-related variables showed no significant relation to supply. The proportion of elderly persons needing LTC was no longer related to change in bed density.

Among the control variables, initial bed density (in 1999) indicating scarcity/competition was significantly negatively associated with bed density under LTCI (at the 1% level). More institutional beds were set up in municipalities with more scarcity, i.e. less competition. Income, an indicator of financial ability, was also significantly negatively associated with supply (at the 1% level). Finally, the growth rate of elderly persons was significantly negatively associated with change in bed density (at the 5% level), probably because of a slower relative increase in supply.

## Discussion

Within the fixed price system of IFs in Tokyo, as hypothesized bed supply was not found to be need-oriented for both overall and additional supply under LTCI. This was also reflected in an inequitable bed distribution. However, the hypothesized cost-dependency was only supported for overall bed supply: overall bed supply was dependent on land price and spatial availability/scale economies, but additional supply was not. The cost-independency of additional supply may be explained by a partial introduction of free pricing for hotel costs for those on higher incomes in 2005, and authorities’ intervention such as supply control and subsidy policy under the LTCI law. For example, local authorities can prevent the establishment of facilities in areas with sufficient beds, and subsidize new facilities in areas with scarce beds. The negative effect of initial bed density and positive effect of subsidy on supply supports this view. Given the positive bivariate association between land price and change in bed density (Additional file 4), which substantially weakened after controlling for initial bed density and/or subsidy, political interventions may have counteracted the cost-dependency of supply. Regarding control variables, the positive association between the proportion of elderly persons and overall supply may be due to the fact that achieving higher ratios of IF beds to elderly inhabitants was a municipal aim under an expansion plan before the introduction of LTCI. This indicates control of authorities on IF bed supply, but without consideration of LTC need.

The second hypothesis regarding orientation of supply (towards both current need and its growth) was supported for the free price system of PNHs in Tokyo. The need-oriented supply of PNHs however did not lead to an equitable bed distribution of PNH (Table 4). As for cost-dependency, the hypothesis was supported for land price as indicator for capital costs, but not for facility size. This may indicate suppliers’ orientation towards scale economies: larger spatial availability encourages suppliers to establish larger facilities under the expectation of economies of scale. Furthermore, supply was negatively related to wages. This unexpected wage-dependency might be attributed to publicly fixed care fees which cover wages, i.e. wages are not relevant to the second hypothesis. Instead, the first hypothesis for cost-dependency in a fixed price system applies and was supported. The fact that wage-dependency is not observed for IFs, for which care fees are also fixed, but for PNHs, might be explained with lower fixed fees at PNHs than IFs [54], and/or stronger cost-consciousness of PNHs. In conclusion, the cost-dependency might constrain the equitable distribution of PNH beds irrespective of need-oriented supply.

As hypothesized, the supply of IFs in NRW as a system of individually bargained prices was fairly independent of costs. Need showed a positive association with overall but not with additional supply after introducing LTCI. This indicates that the orientation of supply towards care need has weakened after LTCI was introduced. In contrast, profit and demand variables were relevant for change in supply. Initial bed density indicating scarcity/competition showed a significant negative association to supply. This implies a market competition after abolishing supply control by authorities. Furthermore, a negative relationship between income and additional supply was found. This finding may reflect a demand generated by the German subsidy system: about one-third of German IF residents are eligible for social assistance [36], and part or all of IF costs are subsidized. For low-income earners, costs do not need to be taken into account when selecting a facility [33]. They are therefore expected to have less incentive to seek cheaper services/facilities and have higher demand for expensive institutional care than middle- or high-income earners who must pay considerable co-payments for institutional LTC [24]. Care need may not play an important role in additional supply since supply met a certain level of need, as only some of those eligible prefer IFs in the given framework. Further analysis is needed to clarify this relationship.

With two cost-relevant variables and none of the need variables being significantly related to bed density, the fixed price system of IF in Japan is more cost-dependent and less need-oriented than the other two systems. Cost-dependent supply might be caused by a price fixed below the equilibrium price, according to economic theory. A mere focus on costs might lead to a need-independent supply and shortage.

The findings of this study are consistent with previous research that showed a negative impact of low fixed prices on supply of IFs [16-18] and small rural hospitals [20,21].

### Limitations

This study has some limitations. First, only two metropolitan areas were compared, in Japan and Germany, which may limit external validity. Second, the analysis was cross-sectional and some assumptions were made to deal with missing data in some years. For example, long-term averages or quasi-medians were used under the assumption that the observed relationships were stable between municipalities over the analysis period. Third, some factors which differ between the markets and may influence supply (like ownership status, utilization of home case service, or other cross-country institutional differences) were not available at the municipal level and could not be incorporated into the analyses. Fourth, bed density in the municipality was used as dependent variable to compare equity in supply/access between the communities. However municipality as proxy is not always identical with community and its bed density does not reflect the distance from facilities. Finally, this study focused on equitable supply, while expenditure, quality and administrative costs for controlling and bargaining are also important outcomes to consider.

### Political implications

Notwithstanding the above limitations, the findings of this study have political implications. Most local authorities in Japanese metropolitan areas have achieved neither total nor equitable supply in the provision of IFs. While the negative impact of costs on bed supply seems to be suppressed by interventions like approval or subsidy policy under LTCI, such measures are seemingly not strong enough to make bed supply and distribution need-oriented. Nevertheless, low fixed price and shortage in supply tend to lower quality [55,56]. Raising prices is therefore desirable to achieve equitable supply in areas of shortage**—**if necessary, under supply control to constrain expenditure [17,18]. Such a change in pricing does not occur automatically [16]. Furthermore, building supra-regional and cost-efficient facilities is not suitable for institutional LTC. As with rural hospitals, easy access within a community is desirable in light of the quality of life of elderly residents. Therefore, regulatory modifications to pricing are required. A system of free prices or individually bargained prices might be alternatives. In this study supply under free pricing was found to be explicitly need-oriented, while orientation towards local supply may have been partly hampered by suppliers’ perception of scale economies. In the system of bargained prices, supply is cost-independent and bed supply is not negatively related to need. The general fear that supply depends predominantly on economic abilities of users in the market-oriented system was not supported in any of the flexible price systems by this study. This study however focuses on geographically equitable supply/access, rather than socioeconomically equitable use that would require different regression models. Flexible pricing probably corrects geographically inequitable access, but might increase fees for institutional care in high-price areas and expand the scope of persons needing financial support. Therefore, additional financial support for low-income (and middle-income, if appropriate) earners should be taken into consideration during a transition to flexible pricing.

## Conclusion

The findings of this study suggest a negative impact of the fixed price system on equitable bed supply in elderly care. The contrasting results for not-fixed price systems expand external validity for the negative impact of fixed price systems, and indicate possible solutions against geographically inequitable supply. The importance of the price system suggests that institutional modifications can be used to achieve equitable distribution of bed supply.

## Endnotes

^a^Scanlon’s model originally refers to the dual market with different prices for private payers and subsidized residents within the same nursing home. In Japan, high income earners are required to pay higher accommodation fees, but unlike in the United States, admission priority is regulated to prevent IF from preferring private payers. Therefore the dual market model as a whole is not suitable for the analysis of Japanese IF.

^b^The sample used includes other similar types of residential care services (17%). 51% of facilities in the sample are located in the three largest metropolitan areas of Tokyo, Osaka and Nagoya.

## Abbreviations

β: Standardized coefficient; CI: Confidence interval; HSFE: Health services for the elderly; IF: Institutional facility; LTA: Long-term average; LTC: Long-term care; LTCI: Long-term care insurance; N, NRW: North Rhine-Westphalia; PNH: Private nursing home; SA: Social-assistance; SCN: Skilled care nurse; SNHE: Special nursing home for the elderly; T: Tokyo.

## Competing interests

The authors declare that they have no competing interests.

## Authors’ contributions

KY: study design, data collection, data analysis and writing. KK: study design and data collection. Both authors read and approved the final manuscript.

## Pre-publication history

The pre-publication history for this paper can be accessed here:

http://www.biomedcentral.com/1472-6963/14/48/prepub

## Supplementary Material

Additional file 1 “A market with a fixed price under equilibrium and a change in supply with increasing Costs (title)” illustrating the general economic theory by means of graphic. Click here for file

Additional file 2 “Correlation matrix for analysis of overall supply of institutional facilities in Tokyo 2010 (title)” presenting the bivariate correlations as supplementary information to multiple regression analysis. Click here for file

Additional file 3 “Correlation matrix for analysis of supply of private nursing homes in Tokyo 2008 (title)” presenting the bivariate correlations as supplementary information to multiple regression analysis. Click here for file

Additional file 4 “Correlation matrix for analysis of change in supply of institutional facilities in Tokyo 2002-2010 (title)” presenting the bivariate correlations as supplementary information to multiple regression analysis. Click here for file
