# Supplementary material for: Impact of a fixed price system on the supply of institutional long-term care: a comparative study of Japanese and German metropolitan areas
Source: BMC Health Serv Res. 2014 Feb 1;14:48. doi: 10.1186/1472-6963-14-48 (PMC3937099; doi:10.1186/1472-6963-14-48)
Supplement: Additional file 1 — “A market with a fixed price under equilibrium and a change in supply with increasing Costs (title)” illustrating the general economic theory by means of graphic. [file 1472-6963-14-48-S1.pdf]

Additional file 1. A Market with a Fixed Price under Equilibrium and a Change in Supply with Increasing Costs

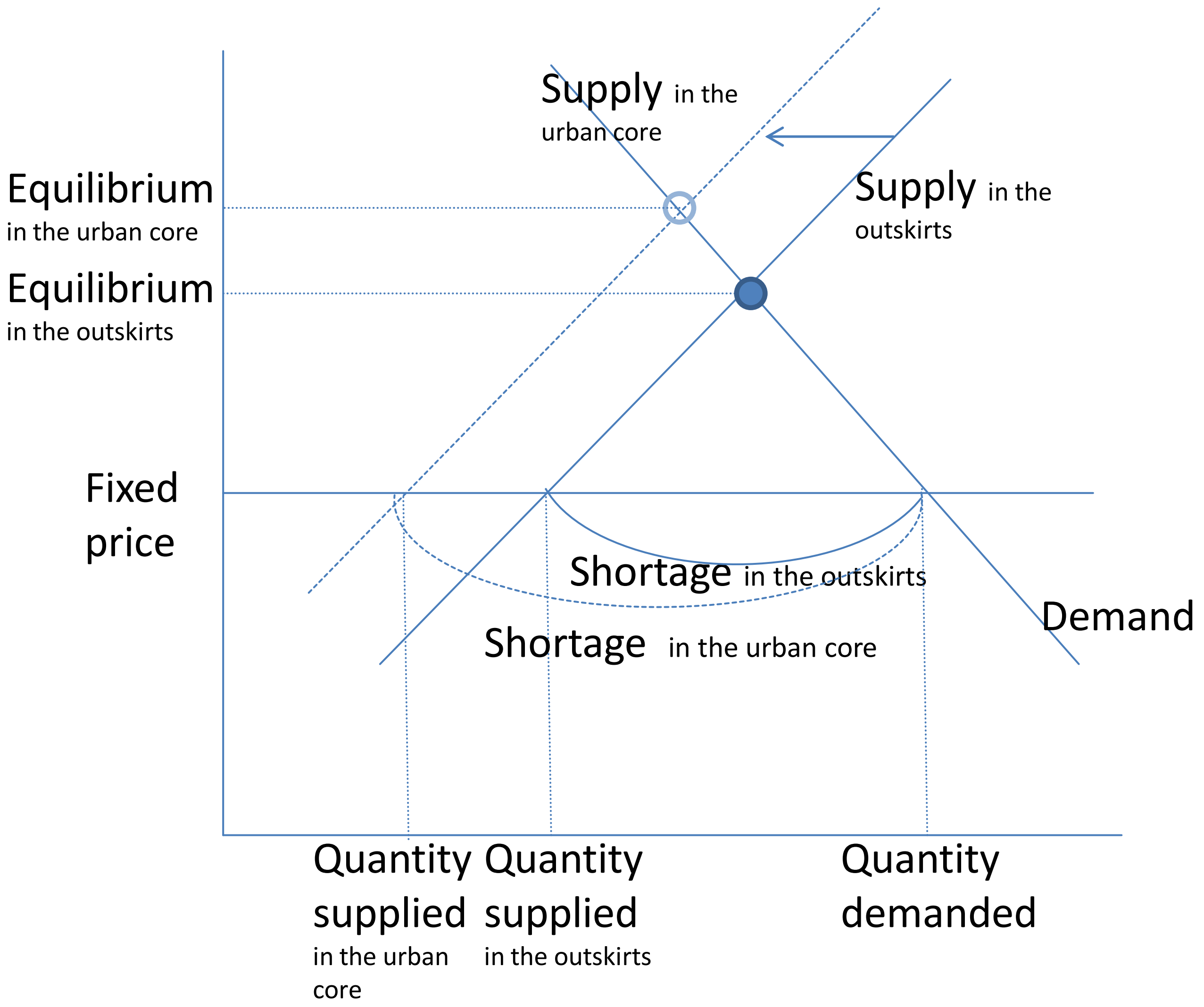

Reference

Wonderling D, Gruen R, Black N: *Introduction to Health Economics*. Maidenhead: Open University Press; 2010.
